# Molecular models of NS3 protease variants of the Hepatitis C virus

**DOI:** 10.1186/1472-6807-5-1

**Published:** 2005-01-21

**Authors:** Nelson JF da Silveira, Helen A Arcuri, Carlos E Bonalumi, Fátima P de Souza, Isabel MVGC Mello, Paula Rahal, João RR Pinho, Walter F de Azevedo

**Affiliations:** 1 Department of Physics, IBILCE/UNESP, São José do Rio Preto, São Paulo, Brazil; 2 Department of Microbiology, Institute of Biomedical Science, USP, São Paulo, São Paulo, Brazil; 3 Adolfo Lutz Institute, São Paulo, São Paulo, Brazil; 4 Department of Biology, IBILCE/UNESP, São José do Rio Preto, São Paulo, Brazil; 5 Center for Applied Toxicology, Butantan Institute, São Paulo, São Paulo, Brazil

## Abstract

**Background:**

Hepatitis C virus (HCV) currently infects approximately three percent of the world population. In view of the lack of vaccines against HCV, there is an urgent need for an efficient treatment of the disease by an effective antiviral drug. Rational drug design has not been the primary way for discovering major therapeutics. Nevertheless, there are reports of success in the development of inhibitor using a structure-based approach. One of the possible targets for drug development against HCV is the NS3 protease variants. Based on the three-dimensional structure of these variants we expect to identify new NS3 protease inhibitors. In order to speed up the modeling process all NS3 protease variant models were generated in a Beowulf cluster. The potential of the structural bioinformatics for development of new antiviral drugs is discussed.

**Results:**

The atomic coordinates of crystallographic structure 1CU1 and 1DY9 were used as starting model for modeling of the NS3 protease variant structures. The NS3 protease variant structures are composed of six subdomains, which occur in sequence along the polypeptide chain. The protease domain exhibits the dual beta-barrel fold that is common among members of the chymotrypsin serine protease family. The helicase domain contains two structurally related beta-alpha-beta subdomains and a third subdomain of seven helices and three short beta strands. The latter domain is usually referred to as the helicase alpha-helical subdomain. The rmsd value of bond lengths and bond angles, the average G-factor and Verify 3D values are presented for NS3 protease variant structures.

**Conclusions:**

This project increases the certainty that homology modeling is an useful tool in structural biology and that it can be very valuable in annotating genome sequence information and contributing to structural and functional genomics from virus. The structural models will be used to guide future efforts in the structure-based drug design of a new generation of NS3 protease variants inhibitors. All models in the database are publicly accessible via our interactive website, providing us with large amount of structural models for use in protein-ligand docking analysis.

## Background

After the development of serological tests for hepatitis A and B viruses in the 1970s it became clear that an additional agent accounted for approximately 90% of transfusion-associated hepatitis (non-A non-B hepatitis, NANBH) [1].

The novel agent, hence termed hepatitis C virus (HCV), currently infects approximately 3% of the world's population and it was classified within the *Flavivirideae *family. Diagnostic tests for anti-HCV antibodies developed thereafter proved that HCV was indeed the predominant cause of NANBH [2]. In view of the lack of vaccines against HCV, there is an urgent need for a treatment of the disease by an effective antiviral drug. This necessity has boosted research on the structural biology of HCV with the primary focus being to identify possible targets for pharmaceutical intervention [3].

Rational drug design has not been the primary way for discovering major therapeutics. However, recent successes in the area give reason to expect that drug discovery projects will increasingly be structure based. One of the possible targets for drug development against HCV is the NS3 protease variants. HCV RNA is translated into a polyprotein that during maturation is cleaved into functional components. One component, nonstructural protein 3 (NS3), is a 631-residue bifunctional enzyme with protease and helicase activities.

The N-terminal portion of the NS3 protein was predicted to contain a serine protease domain as judged from conserved sequence patterns and by homology to Flavi- and Pestiviruses [4-6]. The NS3 serine protease processes the HCV polyprotein by both cis and trans mechanisms. The interative refinement and optimization of drug leads is an effective strategy for generating potent preclinical candidate [7,8]. Ongoing genome sequencing efforts have led to the identification of hundreds of potential therapeutic targets, many of which represent possible sources of crossover pharmacology. Homology or comparative modeling is a key feature of an integrated drug discovery effort because it allows this genomics information to be utilized early in the development of target ligands or in the engineering of ligand specificity [9].

Genome sequencing efforts are providing us with complete genetic blueprints for hundreds of organisms, including humans. We are now faced with assigning, understanding and modifying the functions of proteins encoded by these genomes. This task is generally facilitated by 3D structures [10], which are best determined by experimental methods such as X-ray crystallography and NMR spectroscopy. The theoretical approaches [11] can be divided into physical and empirical methods. The physical prediction methods are based on interactions between atoms and include molecular dynamics and energy minimization [12], whereas the empirical methods depend on the protein structures that have been already determined by experiment. They include combinatorial [13] and comparative modeling [14,15].

Comparative modeling uses experimentally determined protein structures to predict conformation of other proteins with similar amino acid sequences. For modeling of proteins was used restrained-based modeling implemented in the program MODELLER [16]. The models consist of coordinates for all non-hydrogen atoms in the modeled part of a protein. Models are generated entirely automatically in a four-step procedure [17]: (i) fold assignment, (ii) sequence-structure alignment, (iii) model building, and (iv) model evaluation. This procedure was applied to variants of NS3 protease using Perl-CGI, C and MPI programming.

We modeled the structure of variants of NS3 protease variants available in the National Center for Biotchnology Information (Genbank), using structural bioinformatics tools. Knowledge of the three-dimensional structure variants will undoubtedly aid the design of useful inhibitors that may be used as a drug against hepatitis C virus. In order to speed up the modeling process all NS3 models were generated in a Beowulf cluster (BioComp, S.J. Rio Preto, Brazil). The potential of the structural bioinformatics for development of new antiviral drugs is discussed.

## Results and discussion

### Primary sequence comparasion

The identity between the sequences of a bifunctional protease structure (PDB access codes:1CU1, 1DY9) [31,38] (templates) and NS3 protease variants (targets) is shown in Table 1. The secondary structural elements are indicated in the Figure 2 without inhibitor and in the Figure 3 with inhibitor. The sequence from crystallographic structure 1CU1 shows more than 79.1% identity with the sequences of NS3 protease variants, which provide high accuracy for the models (Table 1).

### Quality of the models

The atomic coordinates of crystallographic structure 1CU1 solved to resolution of the 2.5 Å were used as starting model for modeling of the NS3 protease variant structures, and the structure of NS3 complexed with an inhibitor (PDB access code: 1DY9) was used to generate homology models for docking studies. Binding of an inhibitor to the active site of an enzyme is typically connected with local and possibly also global structural rearrangement of the enzyme (induced-fit mechanism). Therefore structure-based drug design preferentially relies on the crystal structures of enzyme-inhibitor complexes containing bound inhibitors of similar chemical structures to the compounds being designed. Such complexes offer more detailed and accurate picture of the inhibitor-enzyme interactions and structural complementarity between the inhibitor and the active site. The homology models of the variants of NS3 protease which used the NS3 complexed with an inhibitor are more adequate to docking simulations. The atomic coordinates of all water molecules were removed from the templates.

The analysis of the Ramachandran diagram φ-ψ plots of the 1CU1 structure (template) were used to compare the overall stereochemical quality of the NS3 protease variants structures against template solved by biocrystallography (Table 1). They present over 94.0% of the residues in the most favorable regions. The same analysis for crystallographic structure (1CU1) present 88.9% of residues in the most favorable, 10.5% additional allowed regions, 0.6% generously allowed regions, and 0.0% disallowed regions, which strongly indicates that the molecular models present good overall stereochemical quality.

### Overall description

The NS3 protease variant structures are composed of six subdomains, which occur in sequence along the polypeptide chain (Figure 2 and 3). The protease domain exhibits the dual β-barrel fold that is common among members of the chymotrypsin serine protease family. The helicase domain contains two structurally related β-α-β subdomains and a third subdomain of seven helices and three short β strands. The latter domain is usually referred to as the helicase α-helical subdomain. The 13-residue protease activation domain of NS4A contributes one strand to the N-terminal protease β-barrel and is considered to be the sixth subdomain [31].

Differences in subdomain structure in the NS3 protease variant molecule and in the structures of the isolated protease and helicase domains were assessed in several ways. Inspection of the molecule revealed that the subdomain folds are similar. Overall preservation of structure is also apparent when the subdomains from the various structures are superposed [31].

The rmsd value of bond lengths and bond angles, the average G-factor and Verify 3D values are shown in Table 2 for NS3 protease variants structures. The same analysis for crystallographic structure (1CU1) present rmsd values of the 0.013Å bond lengths and 1.67°, the average G-fator values of the 0.14 torsion angles and 0.28 covalent geometry, and Verify 3D values of the 321.53 score total and 1.09S quality.

### Database design, access, and interface

**Figure 1:**
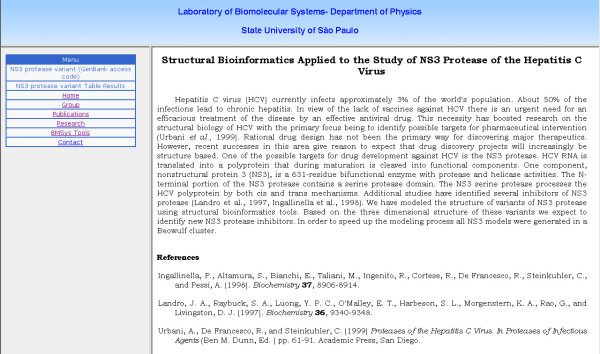
**Database interface. **The database user interface provides user friendly menus. In the menu of main page there are links to NS3 protein variant access codes of the Genbank, which lead to another page with the structural information and analysis about the generated model, such as a small ribbon representation, the atomic coordinates (model and template), and sequence in fasta format (model). The accuracy of the model can be viewed at link "NS3 protease variant table results" which shows results of values of the 3D Profile, rmsd, G-factor, and Ramachandran plot for each model.

**Figure 2:**
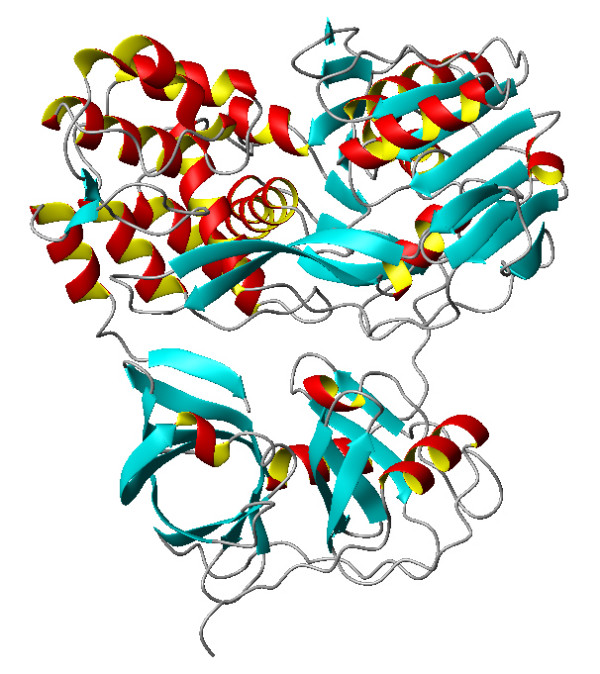
**The strucuture of NS3 protease without inhibitor. **The structure of NS3 protease variant, an engineered molecule that consists of 631 NS3 residues. It has six subdomains: two β barrels in the protease domain (down); two β-α-β subdomains (up on the left) and one α-helical subdomain in the helicase (up on the right). The figure was generated by Molmol [37].

**Figure 3:**
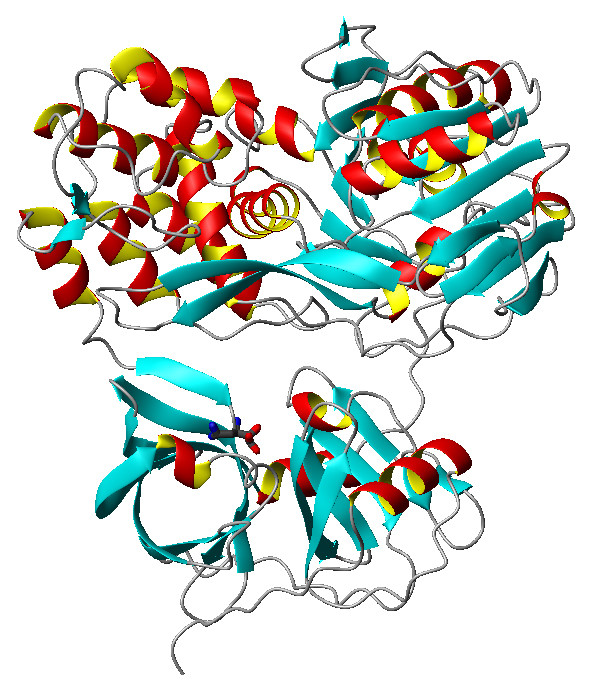
**The strucuture of NS3 protease complexed with 5,5-di-fluoro-2-keto-3-aminopentanoic acid. **The NS3 protease-inhibitor complex shows the interaction between serine protease domain with 5,5-di-fluoro-2-keto-3-aminopentanoic acid. All models were generated using Parmodel [39].

A MySQL database based on relational database management system (RDBMS) was developed to archive protein structure identified in infectious agents such as NS3 protease variants from hepatitis C virus. All supporting data related to the 3D structures modeling, such as protein codes, atomic coordinates in PDB format from modeled proteins, fasta sequence, links to others databases and various information about the protein were arranged in the MySQL [32] database under a master table. The aim this database is to provide access to a collection of annotated models generated by automated homology modelling of NS3 protease variants from hepatitis C virus. All models in the database are publicly accessible via our interactive website (Figure 1) [33]. The database user interface provides user friendly menus, so that all information can be printed in one step from any standard web browser. A small ribbon representation is included to obtain a first impression of the model structure (Figure 2 and 3). Atomic coordinates for the homology models can be downloaded in PDB format and their primary sequence in fasta format. The fields are defined with links to the target sequence, the template structure entries in PDB [34], structural information and analysis. There are two homology models for each sequence in the database, one obtained using 1CU1 as template and other using 1DY9 as template. The second model is adequade for docking simulation, since it was used as template a structure complexed with an inhibitor (PDB access code: 1DY9).

## Conclusions

Large scale protein homology modeling, in which whole sequence databases or whole genomes are used as input into automated modeling algorithms, have been reported by several groups [35]. By utilizing powerful computer systems with multiple processors, these efforts have allowed the creation of large databases of homology models of proteins. This project increases the certainty that homology modeling is an useful tool in structural biology and that it can be very valuable in annotating genome sequence information and contributing to structural and functional genomics from virus, bacteria and other organisms.

Inhibition studies have shown that NS3 is only modestly inactivated by classic serine protease inhibitors such as chloromethylketones or phenylmethyl sylfonylfluoride [36]. The structural models will be used to guide future efforts in the structure based design of a new generation of NS3 protease variants inhibitors. This database is freelly available for all users on the Web, providing us with large amount of structural models for use in protein-ligand docking analysis.

## Methods

### Molecular modeling

Molecular modeling is usually the method of choice when there is a clear relationship of homology between the sequence of a target protein and at least one known structure. This computational technique is based on the assumption that tertiary structures of two proteins will be similar if their sequences are related, and it is the approach most likely to give accurate results [18]. There are two main approaches to homology modeling: (1) fragment-based comparative modeling [14,19] and (2) restrained-based modeling [16]. For modeling of NS3 protease variants from hepatitis C virus we used the second approach. Model building of NS3 protease variants was carried out using the program MODELLER [16]. MODELLER is an implementation of an automated approach to comparative modeling by satisfaction of spatial restraints [20-22]. The modeling procedure begins with an alingment of the sequence to be modeled (target) with related known three-dimensional structure (templates). This alignment is usually the input to the program. The output is a three-dimensional model for the target sequence containing all main-chain and sidechain non-hydrogen atoms.

Next, the spatial restraints and CHARMM energy terms enforcing proper stereochemistry [23] were combined into an objective function. Finally, the model is obtained by optimizing the objective function in Cartesian space. The optimization is carried out by the use of the variable target function method [24] employing methods of conjugate gradients and molecular dynamics with simulated anneling. Several slightly different models can be calculated by varying the initial structure. A total of 1000 models were generated for each enzyme and the final models were selected based on stereochemical quality. All optimization process was performed on a Beowulf cluster with 16 nodes (BioComp, AMD Athlon XP 2100+).

### Analysis of the models

The overall stereochemical quality of the final models for each NS3 protease variants from hepatitis C virus was acessed by the program PROCHECK [25]. The root mean square deviations (rmsd) differences from ideal geometries for bond lengths and bond angles were calculated with X-PLOR [26,27]. G-factor value is essentially just log-odds score based on the observed distributions of the stereochemical parameters. It was computed for the following properties: torsion angles (the analyses provided the observed distributions of φ-δ, χ^1^-χ^2^, χ^-1^, χ^-3^, χ^-4 ^and ω values for each of the 20 amino acid types) and covalent geometry (for the main-chain, bond lengths and bond angles) these average values were calculated using PROCHECK [25]. The Verify-3D measures the compatibility of a protein model with its sequence, these values were calculated using 3D profile [28-30].

## Authors' contributions

NJFS carried out the molecular modeling and participated in the building and design of the database tables, HAA and CEB carried of analysis of the models and created database interface, FPS selected the primary sequences of NS3 protease variants of the hepatitis C virus, IMVGCM participated of the study, PR conceived the study and suggestions in this manuscript, JRRP participated in the study and suggestions in this manuscript, WFA conceived the study, and participated in its analysis and coordination. All authors read and approved the final manuscript.

## Figures and Tables

**Table 1:** Analysis of the Ramachandran plot and identity between template and models of NS3 protease variants. The accuracy of comparative modeling is related to the percentage of sequence identity on which the model is based, correlating with the relationship between the structural and sequence similarities of two proteins. High accuracy comparative models are based on >50% sequence identity to their templates. They tend to have ~1 Å r.m.s. error for the main-chain atoms, which is comparable to the accuracy of a medium-resolution NMR structure or a low-relosution X-ray structure. All structure models in the database were generated using alignments with more than 79% sequence identity, which generating models with high accuracy.

Genbank access code	Residue Range	Identity (%)	Region of the Ramachandran plot (%)			
			Most Favorable	Additional Allowed	Generously Allowed	Disallowed

AJ238800	1027 – 1657	95.0	94.9	5.1	0.0	0.0
AJ238799	1027 – 1657	95.7	95.1	4.6	0.2	0.2
AF139594	1028 – 1658	95.7	95.8	3.8	0.2	0.2
D17763	1033 – 1663	80.5	94.9	4.9	0.2	0.0
AF054247	1027 – 1657	94.0	95.0	4.8	0.2	0.0
AF054248	1027 – 1657	93.8	94.9	5.1	0.0	0.0
AF054249	1027 – 1657	93.8	95.2	4.8	0.0	0.0
D50409	1031 – 1661	80.3	95.1	4.9	0.0	0.0
D84262	1032 – 1662	84.2	95.0	5.0	0.0	0.0
D84263	1025 – 1665	83.1	95.2	4.8	0.0	0.0
D84264	1029 – 1659	83.9	95.2	4.8	0.0	0.0
D84265	1027 – 1657	82.5	94.9	5.1	0.0	0.0
D10749	1027 – 1657	90.5	94.9	5.1	0.0	0.0
D13558	1027 – 1657	94.1	94.8	5.2	0.0	0.0
D14853	1027 – 1657	88.8	95.3	4.7	0.0	0.0
D00944	1031 – 1661	79.8	95.1	4.9	0.0	0.0
D10988	1031 – 1661	79.7	95.1	4.9	0.0	0.0
AF046866	1033 – 1663	80.3	94.7	5.1	0.2	0.0
D49374	1035 – 1665	79.8	94.7	5.1	0.2	0.0
Y11604	1027 – 1657	84.7	95.4	4.6	0.0	0.0
Y13184	1028 – 1658	82.3	95.2	4.8	0.0	0.0
Y12083	1031 – 1661	81.9	94.6	5.2	0.0	0.2
U16362	1027 – 1657	92.6	95.2	4.6	0.2	0.0
U45476	1027 – 1657	94.7	94.9	5.1	0.0	0.0
AJ000009	1027 – 1657	95.2	95.1	4.9	0.0	0.0
M67463	1027 – 1657	89.6	94.9	4.9	0.2	0.0
D63822	1031 – 1661	79.2	95.2	4.8	0.0	0.0
D63821	1032 – 1662	79.1	94.7	5.1	0.2	0.0
D14484	1027 – 1657	94.3	95.8	4.2	0.0	0.0
D11168	1027 – 1657	94.9	95.0	5.0	0.0	0.0
D11355	1027 – 1657	94.6	95.0	5.0	0.0	0.0
D50480	1027 – 1657	93.5	95.0	5.0	0.0	0.0
D50481	1027 – 1657	94.6	95.4	4.6	0.0	0.0
D50482	1027 – 1657	94.7	95.5	4.3	0.2	0.0
D50483	1027 – 1657	93.6	95.2	4.8	0.0	0.0
D50485	1027 – 1657	94.3	95.6	4.4	0.0	0.0
D50484	1027 – 1657	94.1	95.3	4.7	0.0	0.0
D28917	1033 – 1663	80.5	95.2	4.6	0.2	0.0
D30613	1027 – 1657	93.8	95.4	4.6	0.0	0.0
D10934	1027 – 1657	95.2	95.3	4.7	0.0	0.0
AF207762	1027 – 1657	94.0	95.1	4.9	0.0	0.0

**Table 2:** Analysis of the rmsd from ideal geometry, 3D Profile, and G-factor.

Genbank access code	Residue Range	rmsd		3D Profile*		G-factor**	
		Bond Lengths (Å)	Bond Angles (°)	Total Score	S_ideal _Score	Torsion Angles	Covalent Geometry

AJ238800	1027 – 1657	0.020	2.171	299.14	1.03S	0.01	-0.17
AJ238799	1027 – 1657	0.021	2.425	166.68	0.58S	0.07	-0.21
AF139594	1028 – 1658	0.021	2.193	152.89	0.53S	0.09	-0.20
D17763	1033 – 1663	0.019	2.143	290.96	1.00S	0.03	-0.14
AF054247	1027 – 1657	0.020	2.162	288.28	1.00S	0.07	-0.16
AF054248	1027 – 1657	0.020	2.194	292.48	1.01S	0.01	-0.19
AF054249	1027 – 1657	0.020	2.207	290.64	1.00S	0.05	-0.18
D50409	1031 – 1661	0.020	2.188	287.09	0.99S	0.02	-0.17
D84262	1032 – 1662	0.020	2.181	276.59	0.95S	0.06	-0.17
D84263	1025 – 1665	0.020	2.173	283.17	0.98S	0.01	-0.16
D84264	1029 – 1659	0.020	2.174	296.59	1.02S	0.03	-0.15
D84265	1027 – 1657	0.020	2.155	277.67	0.96S	0.02	-0.15
D10749	1027 – 1657	0.020	2.589	287.57	0.99S	0.02	-0.16
D13558	1027 – 1657	0.020	2.157	280.60	0.97S	0.04	-0.15
D14853	1027 – 1657	0.020	2.168	274.13	0.95S	0.02	-0.16
D00944	1031 – 1661	0.020	2.149	287.90	0.99S	0.40	-0.15
D10988	1031 – 1661	0.020	2.171	287.55	0.99S	0.02	-0.16
AF046866	1033 – 1663	0.020	2.957	281.07	0.97S	0.03	-0.16
D49374	1035 – 1665	0.020	2.163	292.23	1.01S	0.02	-0.15
Y11604	1027 – 1657	0.020	2.382	281.93	0.97S	0.02	-0.16
Y13184	1028 – 1658	0.020	2.166	296.66	1.02S	0.02	-0.15
Y12083	1031 – 1661	0.020	2.271	290.58	1.00S	-0.01	-0.21
U16362	1027 – 1657	0.020	2.186	280.49	0.97S	0.01	-0.16
U45476	1027 – 1657	0.020	2.398	281.26	0.97S	0.03	-0.17
AJ000009	1027 – 1657	0.020	2.168	290.86	1.00S	0.03	-0.16
M67463	1027 – 1657	0.020	2.139	281.59	0.97S	0.03	-0.14
D63822	1031 – 1661	0.020	2.385	284.43	0.98S	0.03	-0.16
D63821	1032 – 1662	0.020	2.420	269.09	0.93S	0.03	-0.18
D14484	1027 – 1657	0.020	2.234	285.83	0.99S	0.00	-0.19
D11168	1027 – 1657	0.020	2.160	289.57	1.00S	0.04	-0.15
D11355	1027 – 1657	0.020	2.148	306.49	1.06	0.04	-0.14
D50480	1027 – 1657	0.020	2.123	291.79	1.01S	0.05	-0.13
D50481	1027 – 1657	0.020	2.151	277.60	0.96S	0.06	-0.14
D50482	1027 – 1657	0.020	2.172	284.69	0.98S	0.03	-0.16
D50483	1027 – 1657	0.020	2.149	291.04	1.00S	0.06	-0.15
D50485	1027 – 1657	0.020	2.138	293.16	1.01S	0.03	-0.14
D50484	1027 – 1657	0.020	2.204	289.47	1.00S	0.00	-0.17
D28917	1033 – 1663	0.020	2.221	295.32	1.02S	0.03	-0.19
D30613	1027 – 1657	0.020	2.248	282.94	0.98S	0.02	-0.20
D10934	1027 – 1657	0.020	2.434	289.87	1.00S	0.00	-0.18
AF207762	1027 – 1657	0.020	2.367	308.61	1.07S	0.04	-0.15

*Total Score: is the sum of the 3D-1D scores (statistical preferences) of each residue present in protein. **Ideal Score**: S_ideal _= exp(-0.83+1.008xln(L)); where L is number of amino acids.

**S**_ideal _**Score**: is compatibility of the sequence with their 3D structure. It is obtained Total Score / Ideal Score. S_ideal _Score above 0.45S_ideal_.

**Ideally, scores should be above -0.5. Values below -1.0 may need investigation.
